# Basic Rules of Hygiene Protect Health Care and Lab Workers from Nasal Colonization by *Staphylococcus aureus*: An International Cross-Sectional Study

**DOI:** 10.1371/journal.pone.0082851

**Published:** 2013-12-18

**Authors:** Mitra Saadatian-Elahi, Anne Tristan, Frédéric Laurent, Jean-Philippe Rasigade, Coralie Bouchiat, Anne-Gaëlle Ranc, Gérard Lina, Olivier Dauwalder, Jérôme Etienne, Michèle Bes, François Vandenesch

**Affiliations:** 1 Centre National de Références des Staphylocoques, Hospices Civils de Lyon, Lyon, France; 2 Université de Lyon, Institut National de la Santé et de la Recherche Médicale U1111, Lyon, France; National Institutes of Health, United States of America

## Abstract

Acquisition of nasal *Staphylococcus aureus* (*S. aureus*) colonization by contaminated hands is likely an important determinant of its nasal carriage rate in health care and lab setting. The objective of our cross-sectional study was to assess the prevalence of nasal methicillin-sensitive (MSSA) or -resistant *Staphylococcus aureus* (MRSA) carriage among health care professionals (HCPs) attending an international symposium and to study the association between compliance with hygiene rules, individual-related parameters, and medical conditions with nasal *S. aureus* carriage in this population. After obtaining consent, two nasal swabs were collected. Nasal MSSA and MRSA carriage was measured by the: i) molecular approach targeting *spa*, *mec*A and *mec*A-*orf*X junction sequences, and ii) culture on selective *S. aureus* media combined with *mec*A molecular detection of isolated strains. Information on compliance with hygiene rules, demographic variables, sector of activity and long-term medication was collected by anonymous questionnaire. The participation rate was 32.3%. In total, 176 subjects from 34 countries were included in the analysis. *S. aureus* was isolated from the nasal swabs of 57 (32.4%) subjects, of whom 3 (5.3%) harbored MRSA strains. Overall, 123 subjects reported working in microbiology laboratories with direct manipulation of *S. aureus*, and 29 acknowledged regular contacts with patients. In this exposed population, hydro-alcoholic solutions appeared to have a significant protective effect against nasal *S. aureus* carriage (OR = 0.36; 95% CI: 0.15–0.85). Hospital work was associated with increased risk of nasal *S. aureus* carriage (OR = 2.38; 95% CI: 1.07–5.29). The results of this study showed that compliance with basic rules of hygiene, such as the use of hydro-alcoholic solutions, could reduce the risk of nasal *S. aureus* colonization. Hydro-alcoholic solution could interrupt auto-transmission of the pathogen, consequently decreasing the overall nasal carriage rate, specifically in transient carriers.

## Introduction

*Staphylococcus aureus (S. aureus)*, one of the most prevalent and clinically significant pathogens, causes a broad spectrum of nosocomial and community-acquired infections ranging from benign, superficial skin infections to life-threatening conditions, such as bacteraemia, endocarditis, pneumonia and toxic shock syndrome. The nasal cavity is the main *S. aureus* reservoir, although it can colonize other anatomical sites, such as the skin or intestines [1]. Nasal *S. aureus* colonization is found in 30% of the general population. Nasal carriers have been shown to be at increased risk of acquiring infection [2]. The assumption of a causal relationship between nasal *S. aureus* carriage and infection is supported by studies demonstrating that strains causing the infection are identical to those carried by patients [3], [4].

The mechanisms leading to nasal *S. aureus* colonization are multiple and involve both host (i.e. nasal microbiota, cytokines)- and bacteria (i.e. biofilm, adhesive molecules)-related factors [5]. Advanced age [6], male gender, Caucasian ethnicity [7] and genetic predisposition [8] have been identified as increasing the frequency of colonization. Hormonal contraception [9], medical conditions, such as diabetes [10], haemodialysis [11], human immunodeficiency virus [12], and obesity [13], may also heighten the risk of nasal carriage and consequently augment the risk of infection.

Repeated exposure to *S. aureus* in health care environments has generated the hypothesis that health care professionals (HCPs) could be more frequently colonized. In a cross-sectional study in a teaching hospital, *S. aureus* was isolated from nasal swabs of 43.8% of HCPs, of which 15.2% were methicillin-resistant *S. aureus* (MRSA) [14]. Significant increase in methicillin-sensitive *S. aureus* (MSSA) carriage – from 27% to 46% – has been reported in an epidemiological investigation that compared the prevalence of nasal *S. aureus* carriage among medical students in their third and sixth years of hospital practice [15], supporting the hypothesis that health care work could be a risk factor for nasal carriage. The increased rate of colonization in this population could have implications in nosocomial transmission of the pathogen.

Acquisition of nasal *S. aureus* colonization by contaminated hands is most likely an important determinant of its nasal carriage rate in health care and lab setting. We thought that prevalence of *S. aureus* nasal carriage in health care and lab workers would be similar to those observed in the general population if rules of good laboratory practices and hand hygiene were applied. However, to the best of our knowledge, this has not yet been investigated. The objectives of our study were to: i) evaluate the prevalence of asymptomatic nasal MSSA or MRSA carriage in a relatively homogeneous socio-economical population of otherwise healthy HCPs from different geographic regions (Americas, Asia-Pacific, Africa, Europe, Middle-East) attending an international scientific symposium, and ii) study the association between compliance with hygiene rules, individual-related parameters, and medical conditions with nasal *S. aureus* carriage in this population.

## Methods

### Study population

Individuals attending the International Symposium on Staphylococci and Staphylococcal Infections (ISSSI-Lyon, August 26–30, 2012) were invited to participate. The single exclusion criterion was the presence of medical conditions (wounds, ulcer) contraindicating nasal swab collection. An anonymous questionnaire, focusing on demographic variables, smoking habits, sector of activity, long-term medication, travel, and compliance with hygiene rules in both clinical wards and microbiology laboratories, was completed by study participants.

Two nasal swabs (Copan, Milano, Italy) were collected subsequently from all participants. The first swab was analyzed immediately to determine nasal *S. aureus* carriage and its susceptibility to methicillin, taking a rapid commercial molecular approach based on the use of Xpert SA Nasal Complete® test (Xpert) and GeneXpert® instrument (Cepheid Maurens-Scopont, France). The test consisted of screening for the sequence targets *spa, mec*A genes encoding A protein and 2a penicillin binding protein respectively and *S. aureus orf*X-SCC*mec* junction (SCC). The Xpert test did not allow direct recognition of coagulase-negative staphylococci (CNS) resistant to methicillin (MR-CNS). However, it did provide information on the presence of *mec*A gene encoding resistance to methicillin. A sample negative for *spa* and SCC but positive for *mec*A was considered to contain MR-CNS bacteria.

The second swab was stored at −20°C for further bacterial culture. After cultivation of isolates on ChromAgar Staph aureus® (I2A, Pérols, France), bacterial *S. aureus* DNA was extracted by using QIAprep Spin Miniprep Kit® on QiaCube® instrument (Qiagen, Courtaboeuf, France), according to the manufacturer's recommended protocol. Isolates underwent Alere StaphyType DNA microarray Genotyping (Alere Technologies GmbH, Jena, Germany), as described elsewhere [16]. This microarray covers more than 300 different target sequences, corresponding to approximately 185 distinct genes and their allelic variants. Isolates were assigned to clonal complexes (CCs) by comparing hybridization profiles to previous multilocus sequence typing (MLST) of reference strains in the DNA microarray database [16].

### Ethics consideration

Written informed consent was obtained from all participants. The study was conducted according to national regulations. The study was approved by the local ethics committee (Comité de Protection des Personnes, CPP Sud-Est IV) that considered the study to be non-interventional. Relevant approval regarding access to patient-identifiable information was granted by the French data protection agency (Commission National Informatique et Libertés, CNIL).

### Statistical analysis

Study population characteristics were defined by descriptive analysis. Place of permanent residence was stratified into 7 regions, and sector of activity, into 4 categories. Chi-square and Student T tests were undertaken to compare categorical and quantitative variables, respectively. Prevalence of nasal *S. aureus* carriage with its 95% confidence interval (95%CI) was described. Univariate and multivariate logistic regression models were fitted to assess factors associated with nasal *S. aureus* carriage. The strength of associations was based on crude and adjusted odds ratios (OR) and 95% confidence interval (95% CI). After univariate analysis, all variables with *p*<0.15 were initially included in multivariate models. Backward stepwise analysis was then conducted until all values were p<0.05. All statistical analyses were achieved with Stata 11.0 software. All tests were 2-tailed, with p<0.05 values considered as being significant.

## Results

Of the 550 individuals who attended the conference, 183 (33.3%) participated in the study. Xpert failed with 7 samples, which were excluded, leaving 176 subjects from 34 countries for analysis. Table 1 summarizes the socio-demographic characteristics of the overall study population and the prevalence of nasal *S. aureus* carriage. Of the 176 participants included in the analysis, 100 (56.8%) were women. The prevalence of nasal *S. aureus* carriage was 32.4% (n = 57), of which 5.3% (n = 3) were MRSA strains. All 3 declared direct manipulations of the pathogen; 2 were from Northern Europe, and 1, from Africa. Xpert tests revealed that the prevalence of MR-CNS was 39.6% in the study population, with no differences between nasal *S. aureus* carriers (n = 17, all MSSA) and non-carriers (p = 0.11). Smoking frequency was low and not different between the 2 groups (p = 0.27). The large majority of participants (70.5%) originated from Europe. Twenty-nine subjects declared having moved from their country of birth. In total, 40.3% of participating HCPs were hospital staff and 38.1% worked in the university. Overall, 26 (14.8%) of participants were clinicians, 38 (21.6%) were biologists/microbiologists, 11 (6.3%) were university professors or associate professors, 37 (21.0%) were researchers, 39 (22.2%) were students, and 19 (10.7%) belonged to other categories (industry, government, etc.). A large number of participants (n = 120) reported having travelled out of their country of residence during the 6 months preceding the present study. Long-term treatment for various medical conditions was declared by 68 participants: hormonal contraception (n = 37), non-steroidal anti-inflammatory drugs (n = 11), statins (n = 11), anti-diabetics (n = 8), corticosteroids (n = 6), anti-platelet (n = 4), anti-tumor necrosis factor (n = 3), and others (n = 16). Comparing the socio-demographic characteristics of carriers and non-carriers did not disclose any differences between the 2 groups.

**Table 1:** Characteristics of the study population.

Variable	*S. aureus*	*S. aureus*		Total
	Carriers	Non-carriers	*p-value	Total (n = 176)
	(n = 57)	(n = 119)		(n = 176)
Gender ratio (♀/♂)	0.76	0.89	0.62	0.76
Mean age in years (± SD)	39.9 (±13.2)	40.3 (±12.1)	0.86	40.4 (±12.4)
Mean height in cm (± SD)	174.3 (±10.2)	171.6 (±10.1)	0.10	172.4 (±10.1)
Mean weight in kg (± SD)	71.8 (±16.6)	68.1 (±13.9)	0.12	69.2 (±14.8)
MRSA N (%)	3 (5.3)	0	-	3 (5.3)
MR-CNS N (%)	17 (30.9)	52 (43.7)	0.11	69 (39.6)
Current smoker N (%)	3 (5.3)	12 (10.2)	0.27	15 (8.3)
Region of permanent residence N (%)			0.87	
Western Europe	21 (36.8)	46 (38.6)		67 (38.1)
Southern Europe	0	4 (3.4)		4 (2.3)
Northern Europe	19 (33.3)	34 (28.6)		53 (30.1)
USA, Canada, Latin America	8 (14.0)	16 (13.4)		24 (13.6)
Australia	5 (8.8)	10 (8.4)		15 (8.5)
Asia	1 (1.8)	3 (2.5)		4 (2.3)
Africa	2 (3.5)	3 (2.5)		5 (2.8)
Unknown	1 (1.8)	3 (2.5)		4 (2.3)
Sector of activity N (%)			0.19	
Industry	8 (14.0)	14 (11.7)		22 (12.5)
Hospital	28 (49.1)	43 (36.1)		71 (40.3)
University	19 (33.3)	48 (40.3)		67 (38.1)
Others	2 (3.5)	14 (11.8)		16 (9.1)
Professional category N (%)			0.08	
Physicians/clinicians	7 (12.3)	19 (15.9)		26 (14.8)
Biologists/microbiologists	10 (17.5)	28 (23.5)		38 (21.6)
Professors/associate professors	6 (10.5)	5 (4.2)		11 (6.3)
Scientists/researchers	10 (17.5)	27 (22.7)		37 (21.0)
Students	11 (19.3)	28 (23.5)		39 (22.2)
Others	11 (19.3)	8 (6.7)		19 (10.7)
Unknown	2 (3.5)	4 (3.4)		6 (3.4)
Travel outside country of residence	38 (66.7)	82 (69.5)	0.71	120 (68.6)
Long-term medication N (%)	21 (37.5)	45 (40.2)	0.74	68 (39.1)

*Overall p-value from chi-squared test comparing different categories between carriers and non-carriers

Cultivation of 3 MSSA and 2 MRSA strains failed. Genetic background, i.e. CCs, could therefore be ascertained for 54 MSSA and only 1 MRSA. MSSA-CC30 was the most abundant clone (29.6%), followed by CC15 (18.5%), and CC45 (11.1%). Other frequently-isolated clones were CC5 and CC9 (7.4% each), CC398 and CC7 (5.6% each). CC182, CC1, and CC8 represented less than 2% of isolates. The single MRSA clone was CC22-MRSA-IV. Due to the low number of isolates, we could not analyze the relationship between CC type and variables such as geographical region.

In total, 123 subjects (38 carriers) reported they worked in laboratories with direct manipulation of *S. aureus* on a regular basis (1–3 times per week), and 29 (10 carriers) acknowledged regular contact with patients. These individuals were requested to answer questions on compliance with hygiene rules and laboratory practices. Results were available for 123 persons. The use of gloves, long sleeves, and hydro-alcoholic solutions was reported by almost 75% of this exposed population while 13% declared that they wore dedicated shoes. Among laboratory workers, 52% respected regulations concerning safety cabinets.

Manipulating *S. aureus*, having regular contact with patients and region of permanent residence did not present any association with nasal *S. aureus* carriage (Table 2). We combined 3 sectors of activity (university, industry and others) into 1 group (“non-hospital”), to ascertain the independent role of hospital environment as a risk factor for nasal carriage. In univariate analysis, only hydro-alcoholic solutions showed a significant protective effect against nasal *S. aureus* carriage (OR = 0.42; 95%CI: 0.19–0.96). After multivariate analysis, the protective effect of hydro-alcoholic solutions remained significant (OR = 0.36; 95% CI: 0.15–0.85), and hospital work became a significant factor associated with increased risk of nasal *S. aureus* carriage (OR = 2.38; 95% CI: 1.07–5.29).

**Table 2:** Factors associated with the risk of nasal *S. aureus* carriage in the exposed population.

Variable	Crude OR (95% CI)	p-value	Adjusted OR (95% CI)	p-value
Direct manipulation of *S. aureus*				
No	1			
Yes	0.85 (0.43–1.69)	0.64		
Regular contact with patients				
No	1			
Yes	1.28 (0.54–2.99)	0.58		
Region of permanent residence				
Western Europe	1			
Northern Europe	1.2 (0.57–2.6)	0.60		
USA, Canada, Latin America	1.1 (0.41–2.95)	0.86		
Australia	1.1 (0.33–3.6)	0.88		
Asia	0.73 (0.07–7.4)	0.79		
Africa	1.5 (0.23–9.4)	0.69		
Sector of activity				
Non-hospital	1			
Hospital	1.65 (0.86–3.11)	0.13	2.38 (1.07–5.29)	0.03
Long-term medication				
No	1			
Yes	0.89 (0.46–1.73)	0.74		
**Compliance with hygiene rules**				
Use of safety cabinet (laboratory workers)				
No	1			
Yes	0.86 (0.41–1.81)	0.69		
Use of gloves				
No	1			
Yes	1.32 (0.55–3.17)	0.53		
Use of long sleeves				
No	1			
Yes	0.72 (0.32–1.61)	0.42		
Use of dedicated shoes				
No	1			
Yes	0.39 (0.11–1.44)	0.16		
Use of hydro-alcoholic solutions				
No	1			
Yes	0.42 (0.19–0.96)	0.04	0.36 (0.15–0.85)	0.02

## Discussion

This cross-sectional study showed that working in hospital was associated with increased rate of *S. aureus* nasal carriage and that the use of hydro-alcoholic solutions in subjects exposed to this pathogen in their working environment had a protective effect against nasal colonization. Hydro-alcoholic solutions could interrupt auto-transmission of the pathogen, consequently decreasing the overall nasal carriage rate, specifically in transient carriers. Hand-washing efficacy and the adherence of HCPs to hand hygiene practices are known to be effective in reducing nosocomial infection [17] and MRSA rates in hospital [18] by decreasing cross-transmission. Nevertheless, its impact on auto-transmission after manipulation of the bacterium and leading to nasal colonization has been less investigated. Hands may be contaminated by contact with the pathogen during laboratory procedures or in clinical settings involving direct contact with patients or their environments. Staphylococci are documented to be common laboratory isolates surviving on environmental surfaces and, therefore, touching contaminated surfaces may result in hand acquisition of the pathogen. In hospital settings, hand acquisition of bacterial pathogens could occur after contact with environmental surfaces near hospitalized patients. In a study carried out in a microbiology laboratory, the most contaminated surfaces were computer keyboards and telephone keypads [19]. The authors reported that the use of gloves was associated with higher total bacterial count at the end of work and before hand-washing. Hand-washing at the end of work turned out to be effective in eliminating bacteria. Another study demonstrated MRSA acquisition on gloved hands after contact with environmental surfaces [20]. Gloves are recommended in official guidelines and have been reported to be protective against MRSA acquisition on the hands of laboratory technicians [19] and health care workers. However, guidelines also recommend routine disinfection of hands for better control of hospital-related infections. Some bacteria persisting on the hands after the removal of gloves, could lead to nasal colonization by personal habits, such as nose-picking. In a study of 86 healthy hospital employees, there was a statistically significant correlation between self-reported frequency of nose-picking and both the frequency of positive culture results (p = 0.004) and *S. aureus* load in the nose (p = 0.003) [21]. In addition to the self-protection provided by the use of hydro-alcoholic solutions in HCPs, the impact of the decrease of nasal colonization in HCWs is also of importance in reducing the intra-familial spread of *S. aureus*, as it has been reported that *S. aureus* carriers can “impose” their carrier state upon other household members [22]. Preventing the acquisition of *S. aureus* by family members would contribute to limit staphylococcal infections within the family, which can be seriously complicated if the infecting strain harbors gene encoding for staphylococcal toxins.

It has been proposed that intermittent carriers might actually be non-carriers who carry *S. aureus* only under environmental pressure. Thus, acquisition of nasal colonization by contaminated hands has probably more impact in people classified as “transient carriers” (almost 30% of the general population), as host genetic factors appear to be the predominant determinant of nasal *S. aureus* colonization in about 20% of the population classified as “persistent carriers” [23]. HCPs could become “transient carriers” more often than the general population because of repeated exposure to *S. aureus* in their working environment. Consequently, respect of hygiene rules might contribute to the overall decreased rates of carriage.

Hospitalization has been shown to be a risk factor for both MSSA and MRSA acquisition in patients colonized or not with *S. aureus* at admission [24]. Nevertheless, whether hospital work could be an independent risk factor for nasal MSSA carriage has not been very well explored. In our study, the prevalence of nasal *S. aureus* carriage in hospital workers was 39.4%, and multivariate analysis disclosed that these subjects were at significantly higher risk of nasal *S. aureus* carriage regardless of their professional category. Direct *S. aureus* manipulation was not associated with increased risk of nasal carriage in our study. Compliance with the use of hydro-alcoholic solutions, reported by almost 75% of those manipulating the pathogen, could obviate the potential impact of direct handling of the pathogen on its nasal carriage.

We found that about one-third (32.4%) of the study population were nasal *S. aureus* carriers. This is in range with previously-published studies carried out among attendees of scientific congresses in Europe [25]–[27], employees of clinical microbiology laboratories [28] and the general population [29]–[30]. The results of other studies that investigated the rates of nasal carriage of *S. aureus* in different clinical or non-clinical setting among HCWs are controversial: the prevalence of MSSA carriage among HCPs exceeded that reported in the general population in some studies performed in clinical settings [14], [31], but did not differ significantly from the general population in others [32]. In addition, among hospital staff, high nasal carriage rates were found among nurses, respiratory and laboratory technicians [33]. In a population-based survey conducted in Norway, health care status was not associated with nasal *S. aureus* carriage in the total population. However, female HCPs had 54% greater risk of nasal *S. aureus* carriage (OR 1.54, 95% CI 1.09–2.19) than non-HCPs [34]. A literature review of studies carried out between 1980 and 2006 gave a MSSA prevalence rate of 23.7% (range 0–40%; 95% CI: 10.7–36.7%) among HCPs [35]. The average rate of MRSA carriage was 4.6% (range 0–59%; 95% CI: 1·0–8·2%) [35]. Differences in study design, colonization and antibiotic pressure in the hospitals studied, and lack of information on confounding factors, such as infection control programs in place, limit comparison of these studies and explain, at least partly, the observed discrepancy. Analysis of socio-demographic characteristics by carriage status did not reveal any differences between carriers and non-carriers. The relatively small size of the study population could explain the results.

The population structure of *S. aureus* isolates from our series does not appear to be different from that of other series. Nasal carriage isolates from a collection of 155 carriers (junior medical students, patients and employees of a biomedical facility) sampled in Germany belonged in decreasing order of prevalence to CC8, CC30, CC15 and CC45, which were the most prevalent CCs in our series [16]. Likewise, the population structure of 111 MSSA recovered in Spain from healthy carriers (biology students) revealed that the 4 most prevalent CC (CC30, CC5, CC45 and CC15) were the same as in our series [36]. Of note, we identified 5.6% of isolates belonging to CC398, in accordance with the recently-observed rise of this lineage in both colonizing [37] and invasive isolates [38].

MRSA was identified in 5.3% of *S. aureus* strains, giving a prevalence of 1.6% in the study population. Of note, all 3 MRSA carriers declared that they worked in hospital, providing 4.1% prevalence of MRSA carriage in this sub-category. The rate is comparable to other European studies reporting on MRSA carriage rates in hospital settings [39], but is lower than in the USA [14] or developing countries [40]. Prevalence of MRSA carriage in HCPs varies also according to professional category. In a cross-sectional study in a teaching hospital in the USA, the highest prevalence (9.6%) was among people working in the emergency department [14]. Nurses and nurse aids were at highest risk of being colonized by MRSA in 2 other studies [31], [39]. The most important work-related risk factors for MRSA carriage among HCPs were direct contact with MRSA-infected patients [41]. The small number of MRSA carriers in our study did not allow to assess risk factors and to investigate the effect of compliance with hand hygiene practices on its nasal carriage.

The originality of our study was the collection of data on compliance with good laboratory practices in diverse countries. However, the cross-sectional design demands precaution in interpreting the results. Although we observed a protective effect of hydro-alcoholic solutions against nasal *S. aureus* carriage, the cross-sectional design does not allow establishing causal relationships. Longitudinal studies are needed to determine possible causal relationship between nasal carriage and hand hygiene. Study subjects participated voluntarily and we were able to enroll 32% of the attendees. Selection bias could therefore have occurred if individuals prone to manipulation of the pathogen participated in the study. Due to the anonymous character of the questionnaire, we could not use the congress database to compare the differences in professional and demographic data between participants and non-participants. Moreover, in the absence of longitudinal data, we could not distinguish permanent and transient carriers. Also, inherent to any observational study, there may have been uncontrolled confounders.

In conclusion, prevalence of nasal *S. aureus* carriage in a relatively homogenous socio-economic population of health care and laboratory workers is similar to that encountered in the general population. Compliance with basic rules of hygiene, such as the use of hydro-alcoholic solutions, could reduce the risk of nasal *S. aureus* colonization.
